# “Cold” X5 Hairlaser™ used to treat male androgenic alopecia and hair growth: an uncontrolled pilot study

**DOI:** 10.1186/1756-0500-7-103

**Published:** 2014-02-24

**Authors:** Kenneth Blum, David Han, Margaret A Madigan, Raquel Lohmann, Eric R Braverman

**Affiliations:** 1 Department of Psychiatry and McKnight Brain Institute, University of Florida, College of Medicine, Gainesville, FL, USA; 2 Department of Biostatistics, University of Texas at San Antonio, San Antonio, TX, USA; 3 Department of Nutrigenomics, IGENE, LLC, Austin, TX, USA; 4 Department of Clinical Neurology, Path Foundation NY, New York, NY, USA

**Keywords:** Androgenic alopecia, Distributed laser light, Monochromatic light, Linear regression

## Abstract

**Background:**

Various trials have been conducted on the management and treatment of androgenic alopecia (AGA) or male pattern hair loss using a variety of laser and light sources.

**Methods:**

For this feasibility study, the population was composed of males between the ages of 20 and 60 years who have been experiencing active hair loss within the last 12 months and the diagnosis of AGA. They also had a Norwood-Hamilton classification of 3, 3A, 3 V, 4, 4A, or 5 for the hair thinning patterns and skin type I, II, III, or IV on the Fitzpatrick skin type scale. This two-arm randomized, parallel group study design employed stratifying randomization to balance treatment assignment within three investigational centers with at least 2 subjects enrolled in each Fitzpatrick skin type.

**Results:**

A statistically significant positive trend in hair growth was observed from this pilot study, to evaluate the efficacy of the novel cold X5 hairlaser device for treating male androgenic alopecia. From the repeated measures analysis of variance, difference in mean hair counts over time was statistically significant (F = 7.70; p-value < 0.0001). Subsequent, linear regression of mean hair counts at each time point was performed, and post-hoc analysis found an increasing trend of hair growth over time that was statistically significant (p-value < 0.0001) with the estimated slope of 1.406. Increased hair counts from the baseline to the end of the 26-week period were found to be strongly significant (p-value = 0.0003).

**Conclusion:**

Albeit, sham device failure and resultant missing data from the control group, the positive trend hair growth, was observed due to the chronic use of X5hairlaser device. This positive benefit while in full agreement with other low laser hair devices requires intensive further investigation.

**Trial registration:**

NCT02067260

## Background

### Epidemiology: defining androgenic alopecia

Androgenic alopecia (also known as androgenetic alopecia or *alopecia androgenetica*) is the most common cause of hair loss, and thinning in humans [1]. Androgenic alopecia (AGA) affects an estimated 50 million men and 30 million women in the United States. Genetic predisposition to hereditary hair loss can be inherited from either side of a person’s family or both parents. It is found in men and women of all races and ethnicities. By age 40, forty percent of women and nearly forty percent of men have visible symptoms of hereditary hair loss. By age 50, fifty percent of both genders show signs of the condition. Hair loss is a common and distressing condition. Americans are expected to spend about one billion dollars annually for treatments to combat and cover up hair loss [2,3].

It is well known that both Finasteride® and Minoxidil® are effective treatment methods, but patients who exhibit a poor response to these methods have no additional adequate treatment modalities [4,5]. In this regard, Kim *et al.* reported that after 24 weeks of treatment, a low-level light therapy LLLT group showed significantly greater hair density than the sham device group [6]. In addition Leavitt *et al.*[7] reported that the HairMax LaserComb treatment group showed a significantly greater increase in mean terminal hair density than subjects in the sham device group (p < 0.0001). Moreover, significant improvements in overall hair regrowth were demonstrated in terms of patients' subjective assessment (p < 0.015) at 26 weeks when compared to baseline.

Variants appear in both men and women. However, AGA is also commonly known as male pattern baldness. In males’ classic pattern baldness, hair is lost in a well-defined pattern, beginning above both temples. Hair also thins at the crown of the head. Often a rim of hair around the sides and rear of the head is left. This pattern is dubbed "Hippocratic balding" and may rarely progress to complete baldness. Women do not suffer classic male pattern baldness, instead the hair becomes thinner around the whole scalp, and the hairline does not recede. This is dubbed "female pattern baldness" and may occur in males. This variety of AGA in women rarely leads to total baldness [1,8].

### Androgenic alopecia

More than 95 percent of hair loss in men is caused by AGA. Male pattern baldness is considered a genetic condition, inherited from either the mother or the father's side of the family. However, male pattern baldness also requires the presence of the male hormone testosterone. Genetics cause hair follicles to become sensitive to dihydrotestosterone (DHT), a byproduct of testosterone [9]. The follicles begin to grow smaller, have a shorter life span and eventually fall out altogether or leave behind fuzz.

Various genetic (and possibly environmental-epigenetic) factors apparently play a role in AGA. Although researchers have long studied the factors that may contribute to this condition, many remain unknown.

Recently the existing theories have been challenged on the ground that while the androgens in question are responsible for hair growth on the face and all over the body of men, hair loss only occurs at the top of the scalp. For example, it has been suggested that AGA is a consequence of the anabolic effect of androgens such as hormonal changes leading to structural changes in skin and scalp which in turn cause hair loss [10]. It should be noted, however, that there are as of yet no experiments testing this hypothesis.

### The genetic and hormonal component of male pattern baldness

Much research concerns the genetic component of male pattern baldness, or AGA research indicates that susceptibility to premature male pattern baldness is largely X –linked, which means it is linked to genes on an X-chromosome. Other genes that are not sex linked are also involved. Men whose fathers had experienced hair loss, were 2.5 times more likely to experience hair loss themselves, regardless of the mother's side of the family [11,12]. Large studies in 2005 and 2007 stress the importance of the maternal line in the inheritance of male pattern baldness. German researchers name the androgen receptor gene as a necessary condition for balding [11]. They concluded that a specific variant of the androgen receptor is needed for AGA to develop. This study has been confirmed by other researchers [13]. The androgen receptor gene is recessive. Thus, a female would need two X chromosomes with the defect to show typical male pattern alopecia. Since androgens and their interaction with the androgen receptor are the cause of AGA, the androgen receptor gene plays an important part in its development. There is a plethora of research concerning the role of genes and hormones in the development of male androgenic alopecia and reviewed elsewhere [14-22]. Studies on hormonal imbalance have also been well documented in the literature including concepts involving inhibition of 5-alpha- reductase [23].

### Description of X5 hairlaser

**Figure 1:**
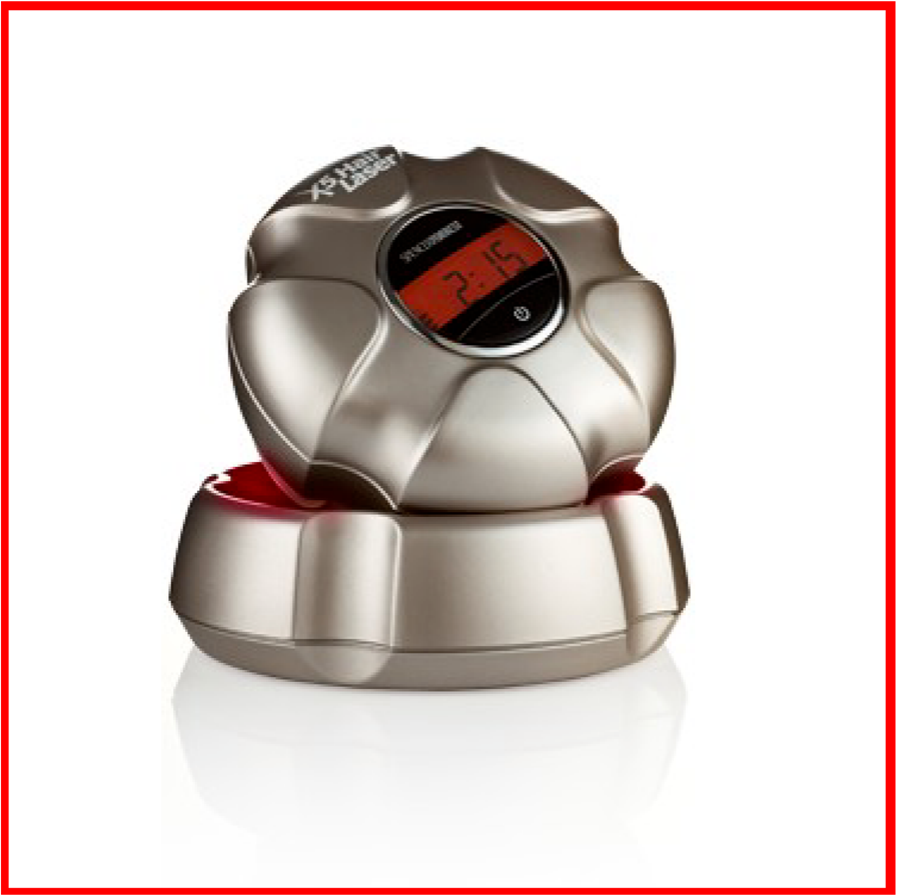
X5 HairLaser.

The X5 HairLaser delivers distributed laser light to the scalp for stimulating hair growth in men diagnosed with AGA. The X5 HairLaser is a precision instrument utilizing cold beam low-level laser light. The X5 HairLaser provides 15 distinct points of laser light to cover an area over nine square inches (23 cm.) of the scalp. Separately molded high quality lenses allow the laser beams to be directed at the scalp directly. The X5 HairLaser provides direct scalp contact since the laser light is delivered by proprietary laser channels, which make direct contact with the scalp. The laser light is not obstructed by existing hair. The X5 HairLaser contains a floating membrane system that insures that light channels conform to the shape of the head and scalp. The X5 HairLaser is a cordless device that contains a rechargeable battery in its base. One charge will supply cordless power for several treatments.The X5 HairLaser has fifteen lasers diodes and fifteen beam-focusing tines. Each tine houses its own laser diode. The fifteen tines are arranged into five groups of three. Each group of three tines moves independently providing perpendicular laser delivery to the user’s scalp. The beam focusing lenses are integrally attached to tines, the tines align the lasers, directly (axially) with the lens. The beam is projected towards the user’s scalp. The tines through which the laser beam is delivered, also part the user’s hair to deliver the laser beam directly to the scalp without obstruction (see Figure 1).

The X5 HairLaser incorporates a colored LCD display that displays the precise elapsed time of the treatment session, as well as the battery power remaining and the charging status of the unit. The X5 HairLaser device is operated by; turning it on, holding the device against the scalp for approximately 15 seconds, then moving it slightly, to a different position on the head. The device is repositioned every 15 seconds in a clockwise or counterclockwise direction. After 10–15 minutes, the session is complete once all of the target areas of the scalp have been given significant exposure to low-level laser light.

### Hypothesis

Based on the literature to date we decided to systematically test the “Cold” X5 Hairlaser™ as a laser device known to deliver distributed laser light to the scalp for stimulating hair growth in men diagnosed with AGA.

## Methods

### Study design

This feasibility study was designed to evaluate the efficacy of the X5 HairLaser, a hand-held, low-level laser device, for the stimulation of hair growth. This study of the X5 HairLaser intended to increase terminal hair growth in males with AGA, was a multi-center, randomized, placebo-controlled study conducted at three sites in the United States. The trial was provided a clinical protocol number by ClinicalTrials.gov Protocol Registration System number NCTO2O67260.

#### Subjects

In this pilot study we relied on the American Academy of Dermatology for hair loss guidelines as part of the inclusion criteria for enrolled patients [24]. The participants who met all entry criteria were randomized in a 2:1 fashion to receive treatment with either the X5 HairLaser or an identical sham device that does not emit laser light. The study population was composed of males between the ages of 20 and 60 years with diagnosis of AGA who had been experiencing active hair loss within the past 12 months. They were also required to have a Norwood-Hamilton classification of 3, 3A, 3 V, 4, 4A, or 5 for the hair thinning patterns and have the skin type I, II, III, or IV on the Fitzpatrick skin type scale (see Inclusion and Exclusion Criteria below). The study was approved by Institution Review Boards (IRB) of the three selected sites. Each subject filled out an approved consent form. Specifically, the IRB was Biomedical Research Alliance of New York (BRANY), 1981 Marcus Avenue Suite 210, Lake Success, New York 11042 (file number 08-02-31-161). The three research locations are listed below the IRB covered all of them: 1) NYU School of Medicine 560 First Avenue, Room 158 New York, NY 10016; (2) Burke Pharmaceutical Research 3633 Central Avenue Suite I, Hot Springs, AR 71913; (3) Hilltop Research 6699 13^th^ Avenue N, St. Petersburg, FL 33710.

#### Inclusion criteria

• Male with Androgenic Alopecia.

• Active hair loss within the last 12 months.

• Good general health.

• Norwood-Hamilton classification of 3, 3A, 3V, 4, 4A or 5.

• Skin Type I, II, III, or IV on the Fitzpatrick Skin Type Scale.

• Has not started any new vitamins or nutritional supplements.

• Continued normal grooming habits.

• Adhering to scheduled office visits in a timely manner.

• Diagnosed according to Guidelines of the American Academy of Dermatology Association.

• Subject must have miniaturized hair present in the target area.

• Subject must be fluent in English.

• Signed an informed consent.

#### Exclusion criteria

• Active malignancy of any type or history of any malignancy, including any malignancy in the treatment area in the past five years.

• History of hypogonadism.

• Subject has used phytotherapy (e.g., saw palmetto) within eight weeks prior to baseline.

• Any active skin infection in the scalp area or scarring in the target area.

• Photosensitivity to laser light.

• Has used Accutane® in the previous year.

• History of poor wound healing.

• History of anticoagulant or antiplatelet use (other than aspirin ≤325 mg., QD, which is stable for three months.

• Has "buzz" cut hairstyle, defined as hair cut to less than one inch in length.

• Has light blond, light gray or white hair.

• Has a chronic dermatological condition (eczema, psoriasis, infection, etc.) of the scalp.

• Has a pacemaker.

• Has had hair transplants, scalp reduction, current hair weave, or tattooing in the target area.

• Has ever received radiation therapy to the scalp, or has had chemotherapy within the past year.

• Has HIV infection, connective tissue disease, a thyroid condition, inflammatory bowel disease.

• Has a history or evidence of drug and/or alcohol abuse within the 12 months prior to Visit 1.

• History or the presence of any serious and/or chronic medical condition(s) [including psychiatric illnesses.

• Has used or currently takes Minoxidil (Rogaine™) during twelve months prior to screening.

• Has taken any of the following medications during the six months prior to screening:

◦ finasteride, (or any other 5á–reductase inhibitor medications),

◦ medications with anti-androgenic properties (e.g., cyproterone acetate, spironolactone, ketoconazole, flutamide, bicalutamide),

◦ topical estrogen, progesterone, tamoxifen, anabolic steroids,

◦ medications which can potentially cause hypertrichosis (e.g., cyclosporine, diazoxide, phenytoin, psoralens),

◦ oral glucocorticoids (inhaled glucocorticoids are permitted), lithium, phenothiazines.

#### Design and subject demographics

A two-arm randomized, parallel group design was employed for this study, stratifying the randomization to balance treatment assignment within 3 investigational centers. Additionally, in the sampling scheme, at least 2 subjects were required to be enrolled in each Fitzpatrick skin type.

A total of 143 study participants were screened on their first visit, based on the inclusion/exclusion criteria and 119 subjects (83.22%) were entered into the study. The diagnosis of male Androgenic Alopecia was made by a trained medical Dermatologist utilizing in many cases modern hair imaging techniques including trichoscan and or trichoscopy.

They were almost uniformly distributed, resulting in approximately 40 subjects enrolled at each of the 3 different study sites. Of those 119 subjects, 70 of them (58.82%) were treated with the X5 hairlaser device while others (41.18%) received the placebo. The study consisted of 5 follow-up visits for the period of 26 weeks. On each visit of the subject, some clinical measurements were taken along with the hair count data which was provided by the imaging vendor, Canfield.

The primary objective of the study was to prove the efficacy and safety of the X5 hairlaser device for treating male AGA through comparison to the control. However, due to a problem in the implementation of the proposed protocol, whereby after evaluation of the entire study the sham device unbeknown to us constituted a laser type lighting effect. This light affected subsequent analysis on this report. While we are cognizant of the impact of the loss of sham control data we decided to conduct pilot data from the treatment group only, excluding the data from the control placebo group. Hence, the focus of this report is to investigate the presence of positive hair growth in the treatment group by examining the trend in hair counts of the 70 treated subjects (viz., a sample of the Intent-to-Treat (ITT) population).

For each subject in this study, the hair counts were supposed to be taken at 6 different time points: Baseline, Week 4, Week 8, Week 14, Week 20, and Week 26. Twenty-two subjects (31.4%) from the treatment group are however missing at least one such measurement from the given dataset due to loss to follow-up or withdrawal from the study, etc. Since it is crucial to have a complete dataset for running a repeated measures analysis of variance (ANOVA) and to test any linear trend over time, data from 48 subjects who completed all study visits with 6 valid hair count measurements were analyzed first in order to confirm any positive trend in the hair growth over time (i.e., a per-protocol analysis). Then, the missing hair counts of 22 remaining subjects with the baseline and at least one valid post-baseline assessment were imputed by carrying the most recent observation from the previous visits to the missing one(s) for each subject (viz., the method of last observation carried forward (LOCF)). The analysis was conducted again on the dataset of all 70 subjects, mixed with the imputed observations.

The brief demographic characteristics of the treatment group are as follows. The age distribution was found to be 47.60 ± 7.82 (mean ± SD) for the partial group of 48 subjects with complete hair counts and 47.04 ± 8.55 for the group of all 70 subjects. There were 7 Hispanic or Latino participants (10.0%) while other 63 (90.0%) were neither Hispanic nor Latino. The race was all white except for one African American and one native Hawaiian (or other pacific islander). The frequency table of the Norwood Hamilton classification of the treated subjects is also shown in the Table 1 below.

**Table 1:** **Frequency distribution of the Norwood Hamilton classification in the treatment group (*n*** **= 70)**

**Class**	**Frequency**
3	7
3A	2
3 V	20
4	19
4A	1
5	21
**Total**	70

We did not systematically rule out other overlapping causes of hair loss but we were confident that the subjects tested had a well-documented diagnosis of male androgenic alopecia.

## Results

### Overall trend of hair growth

With an appropriate aggregation of the classes, none of the variables aforementioned including the distribution of the Fitzpatrick skin type classification and the grouped age variable was found to have a statistically significant association with one another at 5% level of significance.

**Figure 2:**
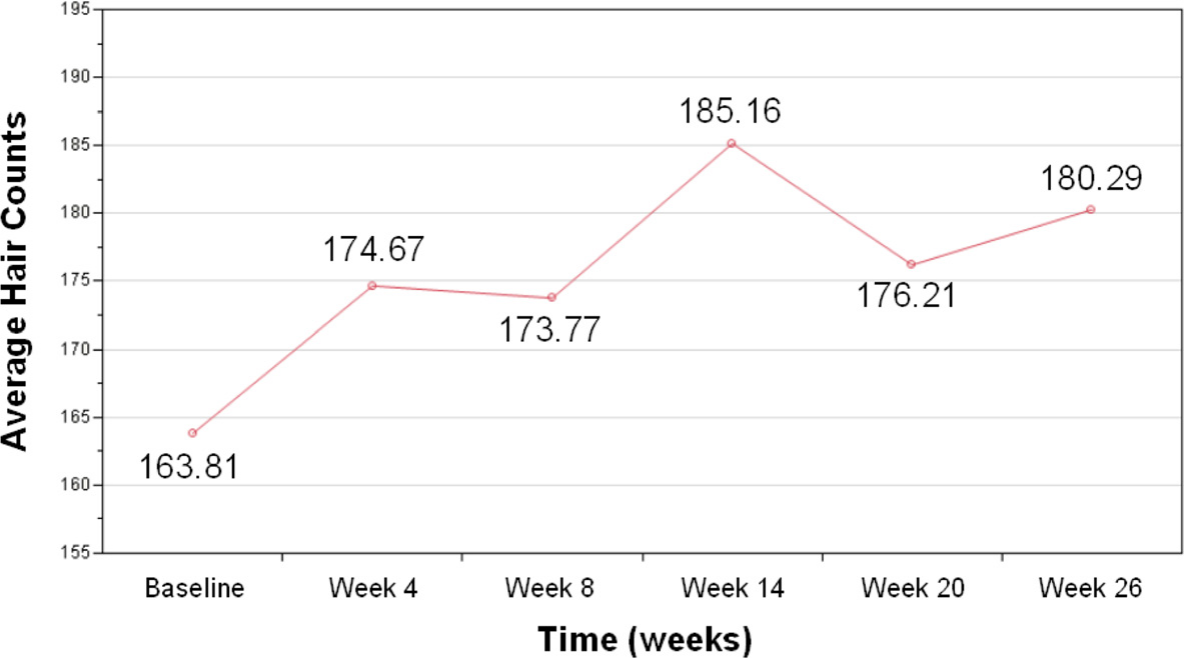
Box-and-whisker plots of the hair counts from the complete data (n = 48).

Table 2 below summarizes the essential sample statistics from the hair counts of 48 subjects with complete observations at each measurement point over the period of 26 weeks. The mean profile was plotted to visualize the results, and it is shown in Figure 2 below, assuming that the measurements were taken in an approximately uniform interval.

**Table 2:** **Sample statistics of the hair counts from the complete data (*n*** **= 48)**

**Sample statistics**	**Baseline**	**Week 4**	**Week 8**	**Week 14**	**Week 20**	**Week 26**
**Minimum**	22.0	37.0	31.0	21.0	26.0	11.0
**25% Percentile**	130.8	144.0	137.0	155.3	144.0	136.5
**Median**	168.0	181.0	168.5	184.5	176.5	183.0
**75% Percentile**	204.5	210.0	217.0	227.5	216.3	229.5
**Maximum**	297.0	302.0	329.0	335.0	311.0	321.0
**Mean**	163.80	174.70	173.80	185.20	176.20	180.30
**Standard deviation**	65.60	61.87	71.32	69.90	68.43	73.05
**Standard error**	9.47	8.93	10.29	10.09	9.88	10.54
**95% Confidence interval**	(144.8, 182.9)	(156.7, 192.6)	(153.1, 194.5)	(164.9, 205.5)	(156.3, 196.1)	(159.1, 201.5)

**Figure 3:**
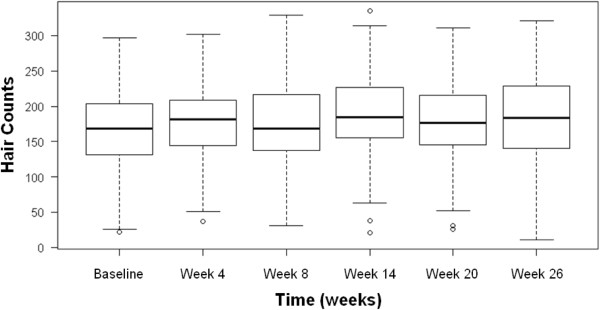
**Box-and-whisker plots of the hair counts from the complete data (n = 48).** (Empty dots indicate the outliers from each measurement group).

From Figure 2, an overall increasing-trend is apparent. The amount of variability is also increasing at each measurement point (as shown in the comparative box-and-whisker plots of Figure 3 above). From the repeated measures analysis of variance, it was shown that the difference in mean hair counts over time is statistically significant (F = 7.70; p-value < 0.0001). Subsequently, a linear regression on the mean hair counts at each measurement time point was performed as a post-hoc analysis, and it was found that a systematically increasing trend of hair growth over time is statistically significant (p-value < 0.0001) with the estimated slope of 1.406. However, the R^2^ value of 0.0050 indicated the presence of a large amount of variability which cannot be explained by the duration of the device usage only. For this reason, it was not successful to model the trend in the hair counts over time using a growth curve approach. When the end points alone were considered, an increase in the hair counts from the baseline to the end of the 26-week period was nevertheless found to be strongly significant (p-value = 0.0003).

Consistent results were obtained using the mixed dataset of 70 subjects with some imputed values. Table 3 summarizes the sample statistics calculated from this mixed dataset of hair counts at each measurement point over the period of 26 weeks. Again from the repeated measures analysis of variance, the change in mean hair counts over time was shown to be statistically significant (F = 10.60; p-value < 0.0001). From the post-hoc analysis, a linear regression on the mean hair counts at each measurement time point revealed the statistically significant presence of a linearly-increasing trend of the hair growth over time (p-value < 0.0001) with the slightly smaller slope estimate of 1.355. Again, a very low R^2^ value of 0.0047 indicated the presence of a large amount of variability.

**Table 3:** **Sample statistics of the hair counts from the mixed data (*n*** **= 70)**

**Sample statistics**	**Baseline**	**Week 4**	**Week 8**	**Week 14**	**Week 20**	**Week 26**
**Minimum**	22.0	33.0	22.0	21.0	26.0	11.0
**25% Percentile**	121.0	125.3	130.8	137.3	135.3	129.5
**Median**	164.0	172.0	165.0	182.5	172.0	181.5
**75% Percentile**	201.5	203.5	214.8	224.5	214.3	225.3
**Maximum**	303.0	302.0	329.0	335.0	311.0	321.0
**Mean**	159.00	167.90	169.30	178.90	170.00	174.80
**Standard deviation**	65.39	63.55	71.24	69.63	67.46	68.97
**Standard error**	7.82	7.60	8.52	8.32	8.06	8.24
**95% Confidence interval**	(143.4, 174.6)	(152.7, 183.1)	(152.4, 186.3)	(162.3, 195.5)	(153.9, 186.1)	(158.4, 191.2)

### Trend of hair growth by Age group

**Figure 4:**
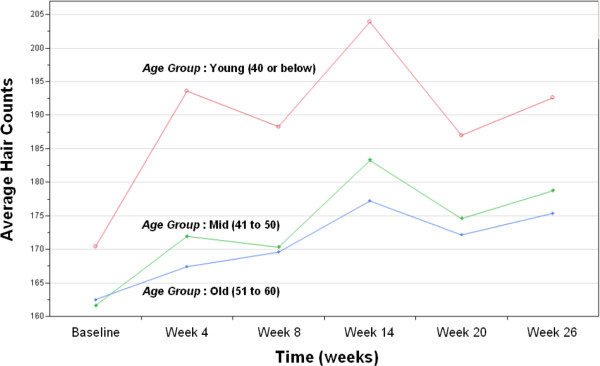
**Mean profile plots of the hair counts from 3 age groups of the complete data (*n*** **= 48).**

In order to examine any differential effect of the device on the hair growth trend of different age groups, the treated subjects were grouped into 3 different classes: (a) Young (40 or below), (b) Mid (between 41 to 50 inclusive), and (c) Old (between 51 to 60 inclusive). The 3 classes were chosen such that each holds an approximately uniform number of observations from the dataset. Table 4 below provides the sample statistics of the hair counts from each of these age groups formed from the dataset of 48 subjects with complete observations. Figure 4 describes the mean profile plots of the hair counts of these age groups over the period of 26 weeks. From Figure 3, a similar overall increasing pattern is observed among the 3 age groups. Although the Young group had higher average hair counts at the baseline compared to the Mid or Old groups, the increments of hair counts by the end of the 26-week period were quite comparable among all 3 groups. All three age groups shared remarkably similar mean profiles, but the pattern of the Young group, was observed to be more erratic, with a larger variation, while the Mid and Old groups exhibited similar and consistent trends of increasing hair counts over time.

**Table 4:** **Sample statistics of the hair counts from 3 age groups of the complete data (*n*** **= 48)**

**Sample statistics**	**Baseline**	**Week 4**	**Week 8**	**Week 14**	**Week 20**	**Week 26**
**(a)** Age group: Young (40 or below) (*n* = 10)						
**Minimum**	26.0	89.0	33.0	63.0	74.0	64.0
**25% Percentile**	119.8	137.8	105.0	114.8	87.8	85.5
**Median**	191.0	198.0	220.5	224.0	202.0	227.0
**75% Percentile**	224.8	249.3	261.0	264.8	257.8	239.5
**Maximum**	280.0	279.0	286.0	292.0	311.0	321.0
**Mean**	170.40	193.60	188.30	203.90	187.00	192.60
**Standard deviation**	81.78	62.11	87.63	79.59	83.69	85.27
**Standard error**	25.86	19.64	27.71	25.17	26.46	26.96
**95% Confidence Interval**	(111.9, 228.9)	(149.2, 238.0)	(125.6, 251.0)	(147.0, 260.8)	(127.1, 246.9)	(131.6, 253.6)
**(b)** Age group: Mid (between 41 and 50 inclusive) (*n* = 19)						
**Minimum**	43.0	51.0	42.0	38.0	26.0	11.0
**25% Percentile**	138.0	155.0	152.0	159.0	157.0	147.0
**Median**	165.0	182.0	166.0	186.0	178.0	180.0
**75% Percentile**	194.0	207.0	208.0	219.0	204.0	228.0
**Maximum**	269.0	262.0	272.0	259.0	265.0	264.0
**Mean**	161.60	171.90	170.30	183.30	174.60	178.70
**Standard deviation**	54.98	53.10	56.82	55.01	58.58	62.84
**Standard error**	12.61	12.18	13.03	12.62	13.44	14.42
**95% Confidence interval**	(135.1, 188.1)	(146.4, 197.5)	(142.9, 197.7)	(156.7, 209.8)	(146.3, 202.8)	(148.5, 209.0)
**(c)** Age group: Old (between 51 to 60 inclusive) (*n* = 19)						
**Minimum**	22.0	37.0	31.0	21.0	31.0	29.0
**25% Percentile**	109.0	114.0	110.0	126.0	133.0	114.0
**Median**	169.0	164.0	159.0	175.0	168.0	157.0
**75% Percentile**	203.0	203.0	206.0	196.0	203.0	206.0
**Maximum**	297.0	302.0	329.0	335.0	308.0	320.0
**Mean**	162.50	167.40	169.60	177.20	172.20	175.40
**Standard deviation**	69.56	70.61	77.89	79.36	72.17	78.97
**Standard error**	15.96	16.20	17.87	18.21	16.56	18.12
**95% Confidence interval**	(129.0, 196.1)	(133.4, 201.5)	(132.0, 207.1)	(139.0, 215.5)	(137.4, 206.9)	(137.3, 213.4)

Table 5 below summarizes the results of formal statistical tests to detect the mean difference of hair counts across the measurement time points by the repeated measures analysis of variance. The tests were performed for each of the 3 age groups, and once the results were found to be significant, the post-hoc analysis of a linear trend over time was conducted.

**Table 5:** Test results for a linear trend of the hair growth for each age group

**Dataset**	**Age group**	**Sample size (*n*)**	**Repeated measures ANOVA**		**Post-hoc linear trend test**		
			***F***	***p*-value**	***p*-value**	**Slope**	***R***^**2 **^**value**
**Complete data (*n*** **= 48)**	Young	10	1.65	0.1663	–	–	–
	Mid	19	3.76	0.0039	0.0013	1.520	0.0086
	Old	19	3.24	0.0098	0.0009	1.229	0.0033
**Mixed data (*n*** **= 70)**	Young	15	2.66	0.0295	0.1054	1.215	0.0036
	Mid	29	5.12	0.0002	0.0004	1.337	0.0066
	Old	26	5.38	0.0002	< 0.0001	1.455	0.0046

**Figure 5:**
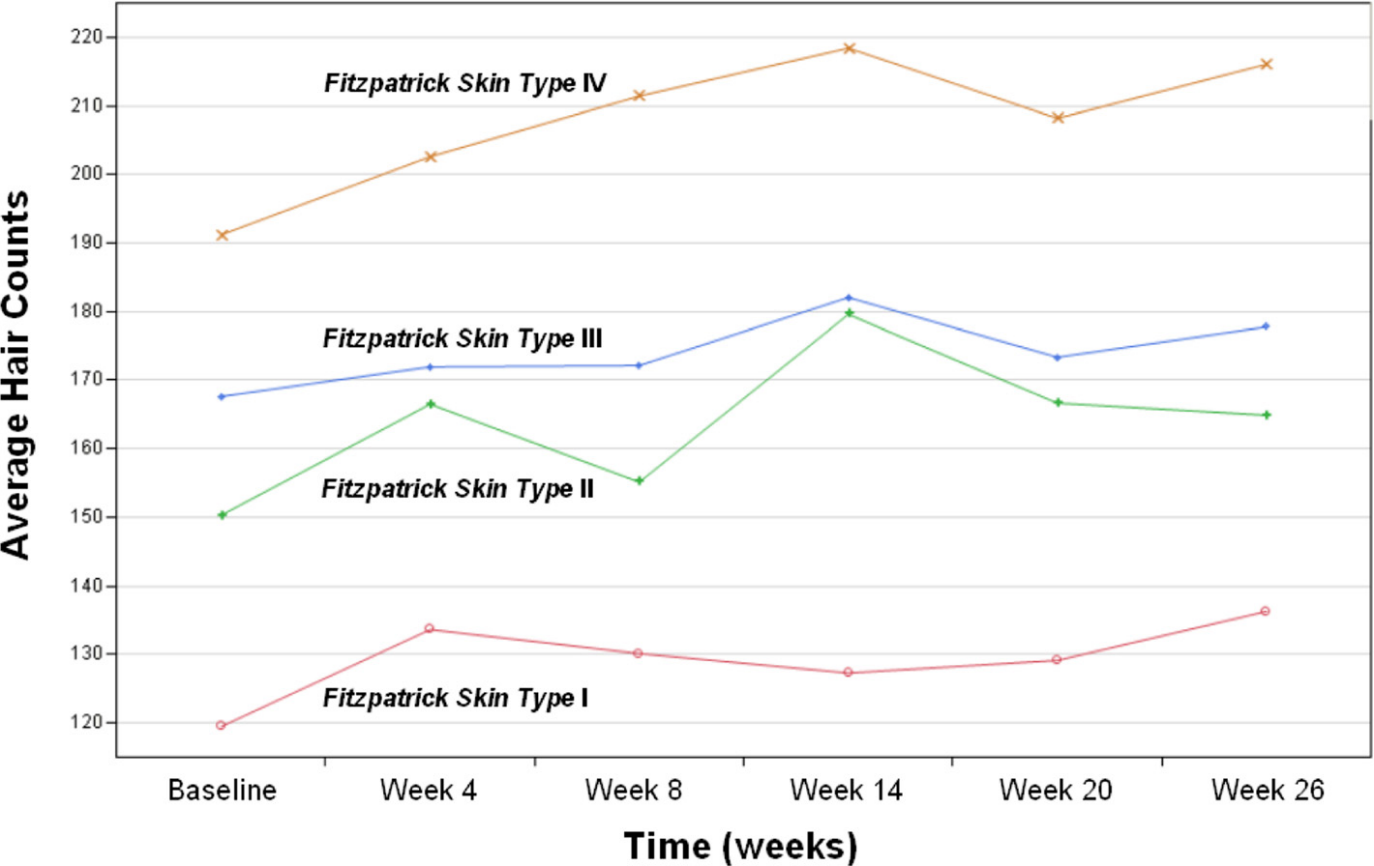
**Mean profile plots of the hair counts from 4 Fitzpatrick skin types of the complete data (*n*** **= 48).**

Between the complete dataset of 48 subjects and the mixed dataset of 70 subjects, the results of analysis were largely consistent. Although the imputation helped to capture the marginal significance of the mean difference of hair counts over time for the Young group (p-value = 0.0295), a linear trend of the hair growth was still not detected (p-value = 0.1054) from the post-hoc analysis as in the complete data case. This may be due to a smaller sample size allocated for the group Young, resulting in higher variability and a more erratic profile as shown in Figure 3. Although the graphical trends are similar across the groups, it seems that the older groups exhibit slightly more consistent and stronger linear trend of the hair growth over time Figure 5. As pointed out earlier though, low R^2^ values indicated the presence of a large amount of variability which cannot be explained by the duration of the device usage only.

### Trend of hair growth by skin type

To determine the presence, of any effect of the device on the hair growth trends that was dependent on skin-type, the subjects were divided into 4 groups according to their Fitzpatrick skin types (1 being the most sensitive and IV being the least sensitive). Each class held an approximately uniform number of observations from the dataset. Table 6 below summarizes the sample statistics of the hair counts in each of these skin types from the complete dataset of 48 subjects. Figure 5 shows the mean profile plots of the hair counts of these 4 skin types over the period of 26 weeks. The more sensitive the skin type is; the less average hair counts are, at the baseline from Figure 5. It was also interesting to observe that among the 4 skin types, the type IV exhibits the strongest pattern of hair counts increasing linearly over time, and this was statistically confirmed by the test results shown in Table 7.

**Table 6:** **Sample statistics of the hair counts from 4 Fitzpatrick skin types of the complete data (*n*** **= 48)**

**Sample statistics**	**Baseline**	**Week 4**	**Week 8**	**Week 14**	**Week 20**	**Week 26**
**(a)** Fitzpatrick skin type I (*n* = 6)						
**Minimum**	22.0	37.0	31.0	21.0	26.0	11.0
**25% Percentile**	37.8	51.3	46.0	33.8	29.8	24.5
**Median**	119.0	119.0	131.0	124.5	133.5	139.5
**75% Percentile**	197.0	213.8	199.8	217.0	203.8	242.3
**Maximum**	227.0	279.0	256.0	250.0	269.0	264.0
**Mean**	119.50	133.70	130.20	127.30	129.20	136.30
**Standard deviation**	83.47	94.21	87.43	94.15	96.38	107.30
**Standard error**	34.08	38.46	35.69	38.44	39.35	43.82
**95% Confidence interval**	(31.9, 207.1)	(34.8, 232.5)	(38.4, 221.9)	(28.5, 226.1)	(28.0, 230.3)	(23.7, 249.0)
**(b)** Fitzpatrick skin type II (*n* = 13)						
**Minimum**	26.0	89.0	33.0	63.0	74.0	64.0
**25% Percentile**	104.5	116.5	101.5	120.0	121.0	109.0
**Median**	150.0	180.0	156.0	185.0	157.0	163.0
**75% Percentile**	192.0	196.0	185.5	223.0	200.5	213.5
**Maximum**	297.0	302.0	329.0	335.0	308.0	313.0
**Mean**	150.30	166.50	155.20	179.60	166.70	164.90
**Standard deviation**	72.79	56.90	71.08	70.25	62.89	69.72
**Standard error**	20.19	15.78	19.71	19.49	17.44	19.34
**95% Confidence interval**	(106.3, 194.3)	(132.2, 200.9)	(112.2, 198.1)	(137.2, 222.1)	(128.7, 204.7)	(122.8, 207.1)
**(c)** Fitzpatrick skin type III (*n* = 15)						
**Minimum**	48.0	51.0	42.0	77.0	52.0	75.0
**25% Percentile**	138.0	144.0	137.0	149.0	143.0	133.0
**Median**	166.0	167.0	166.0	177.0	168.0	163.0
**75% Percentile**	214.0	211.0	217.0	226.0	224.0	226.0
**Maximum**	280.0	274.0	309.0	314.0	304.0	320.0
**Mean**	167.70	172.00	172.20	182.10	173.40	177.80
**Standard deviation**	62.89	59.82	67.89	64.64	67.00	71.35
**Standard error**	16.24	15.45	17.53	16.69	17.30	18.42
**95% Confidence interval**	(132.8, 202.5)	(138.9, 205.1)	(134.6, 209.8)	(146.3, 217.9)	(136.3, 210.5)	(138.3, 217.3)
**(d)** Fitzpatrick skin type IV (*n* = 14)						
**Minimum**	128.0	142.0	130.0	156.0	140.0	153.0
**25% Percentile**	163.0	160.0	155.5	179.5	171.5	179.3
**Median**	185.5	197.0	211.5	200.0	196.0	211.0
**75% Percentile**	225.8	244.8	269.0	265.5	256.0	244.8
**Maximum**	269.0	268.0	286.0	292.0	311.0	321.0
**Mean**	191.20	202.60	211.40	218.40	208.20	216.10
**Standard deviation**	42.61	43.48	55.40	49.19	51.44	48.74
**Standard error**	11.39	11.62	14.81	13.15	13.75	13.03
**95% Confidence interval**	(166.6, 215.8)	(177.5, 227.7)	(179.4, 243.4)	(190.0, 246.8)	(178.5, 237.9)	(187.9, 244.2)

**Table 7:** Test results for a linear trend of the hair growth for each skin type

**Dataset**	**Fitzpatrick skin type**	**Sample size (*n*)**	**Repeated measures ANOVA**		**Post-hoc linear trend test**		
			***F***	***p*-value**	***p*-value**	**Slope**	***R***^**2 **^**value**
**Complete data (*n*** **= 48)**	I	6	0.61	0.6897	–	–	–
	II	13	3.16	0.0135	0.0464	1.400	0.0053
	III	15	1.54	0.1879	–	–	–
	IV	14	4.62	0.0011	0.0003	2.114	0.0229
**Mixed data (*n*** **= 70)**	I	7	0.66	0.6599	–	–	–
	II	20	5.19	0.0003	0.0006	1.791	0.0083
	III	27	2.49	0.0346	0.0046	0.953	0.0025
	IV	16	4.73	0.0008	0.0004	1.846	0.0169

Table 7 above summarizes the results of statistical tests to detect the mean difference of hair counts across the measurement time points by the repeated measures analysis of variance. Just like in the analysis of the age groups, the tests were performed for each of the 4 skin type groups, and once the results were found to be significant, the post-hoc analysis of a linear trend, over time was conducted. Between the complete dataset of 48 subjects and the mixed dataset of 70 subjects, the results of analysis were again largely consistent even though the imputation captured the marginal significance of the mean difference of hair counts over time for the skin type III (*p*-value = 0.0346). The skin type I, had the least number of subjects assigned in both cases, and it did not show any significant mean difference of hair counts nor a linear trend over time. Although not clear, it seems that the least sensitive skin type responds the best to the device for the hair growth over time. Again, low *R*^2^ values indicated the presence of large variability which cannot be accounted for by the duration of the device usage only.

## Discussion

While this multi-centered study was originally planned as a randomized –sham –controlled double-blinded clinical study, it was determined following the investigation as planned, that the sham device was emitting hair growth light that ruined the control data and subsequent results. However, the present results provide significant impetus to develop a better sham device and design a clinical study in a larger cohort.

With that stated, this pilot study to evaluate the efficacy of the cold X5 hairlaser device for treating male AGA resulted in a statistically significant positive trend, in hair growth. From the repeated measures analysis of variance, it was shown that the difference in mean hair counts over time is statistically significant (F = 7.70; p-value < 0.0001). Subsequently, a linear regression on the mean hair counts at each measurement time point was performed as a post-hoc analysis, and it was found that a systematically increasing trend of hair growth over time is statistically significant (p-value < 0.0001) with the estimated slope of 1.406. The increase in hair counts, at the end points, from the baseline to the end of the 26-week period, was nevertheless, found to be strongly significant (p-value = 0.0003). As stated earlier to confirm that this effect was caused by the device and not by a random chance, a more systematic comparative study should be conducted with an appropriate control.

The main limitation of this investigation is the inability to show the difference between the device and an appropriate sham control. The investigation is also limited by the size of the population studied. In future studies we intend to increase our population since this is a non-invasive procedure. However, we are encouraged that because of the stringent inclusion/exclusion criteria these potentially important results will be confirmed in necessary larger –controlled clinical trials, in the future.

The short-term safety of the device was ensured since during the 26 weeks of this study, no any clinically severe adverse events were associated with the X5 hairlaser device.

While we cannot detail the mechanism of action of the X5 (see below) the device was designed to deliver low level laser light directly to the scalp bypassing any intervening hair. The light is delivered by light tines (15 light pipes mounted on five tripods) so that the device conforms to the contour of the scalp and makes direct contact. Other devices simply direct beams into the hair and scalp at angles that cannot be precisely controlled.

Moreover, this low-level laser therapy is intended to bio-stimulate. The effects are biochemical not thermal because the low power nature of low-level laser’s, cannot cause heating damage to living tissue. Lasers are of two principal types, "hot" and "cold", and they are distinguished by the amount of peak power they deliver. "Hot" lasers deliver power up to thousands of watts. They are used in surgery because they can make an incision that is very clean with little or no bleeding and because the laser cauterizes the incision as it cuts. They are also used in surgery that requires the removal of unhealthy tissue without damaging the healthy tissue that surrounds it.

"Cold" lasers such as the X5 HairLaser produce a lower average power of 100 milliwatts or less. This is the type of laser that is used for therapeutic purposes and it is typically, although not always, pulsed. The light is on for only a fraction of a second because it is pulsed, (turned on and off) at so many pulses per second. Pulsation results in an average power output that is very low compared to the maximum or peak output. Therefore, most therapeutic lasers produce a high peak but low average power output. Therapeutic laser is low-level laser therapy (LLLT) with photochemical rather than thermal effects [25-28]. The light is either visible (red), in most cases or invisible (infrared).

### Proposed mechanism of action

Energy is transferred in the form of photons [25]. Photons are transmitted through the skin's layers (the dermis, epidermis and the subcutaneous tissue or tissue fat under the skin). Light waves in the near infrared ranges penetrate deeper than all light waves in the visible spectrum. Photons enter the tissue and are absorbed in the mitochondria and at the cell membrane [26]. The photon energy is converted to chemical energy within the cell in the form of adenosine triphosphate (ATP) [27]. Light emitting diode (LED) in the red region and low-level laser in the near infrared region correspond well with the characteristic energy and absorption levels of the relevant components of the respiratory chain. This LED and Laser stimulation vitalizes the cell by increasing the mitochondrial ATP production [28].

It is believed that successful laser therapy treatment starts with the stimulation of cell functions by the laser light. Research has shown that mitochondria are sensitive to irradiation with monochromatic light [29]. Irradiation with light at a wavelength of 650 nm enhances ATP synthesis [28]. The increase in ATP is believed to be one of the underlying mechanisms that help to stimulate hair follicles [30].

Although it is difficult to ascribe an accurate mechanism of action of the “Cold” X5L, the hypothesis, that irradiation of the scalp with this hair laser device will result in a statistically significant increase in hair growth as compared to a control, is supported by the combination of mechanisms that increase both ATP production and microcirculation.

## Conclusions

While there are number of drugs and even other techniques to induce hair follicle growth alternatives are still important [31-33]. Albeit, sham device failure and resultant missing data from the control group, the positive trend hair growth due to the chronic use of X5hairlaser device was observed. This potential positive benefit is in full agreement with other low laser hair devices and following required larger confirmatory studies should have clinical utility for treating male AGA.

## Competing interests

Kenneth Blum, PhD is a paid consultant and received remuneration from Mark Kress. There are no other financial conflicts.

## Authors’ contributions

KB wrote the initial draft of the manuscript and coordinated the statistical analysis; DH performed all the required statistical analysis and developed the methodology involved in the presentation of results; MAM assisted in writing portions of the manuscript and assisted in all aspects of the writing and edits; RL provided required literature citations; ERB provided literature citations and clinical expertise. All authors read and approved the final manuscript.
